# Commentary: Tracing Management and Epidemiological Characteristics of COVID-19 Close Contacts in Cities Around Chengdu, China

**DOI:** 10.3389/fpubh.2022.913189

**Published:** 2022-07-08

**Authors:** Ali Cheshmehzangi, Tong Zou, Weixuan Chen, Hengcai Chen, Zhaohui Su

**Affiliations:** ^1^Network for Education and Research on Peace and Sustainability (NERPS), Hiroshima University, Hiroshima, Japan; ^2^Department of Architecture and Built Environment, University of Nottingham Ningbo China, Ningbo, China; ^3^Center on Smart and Connected Health Technologies, Mays Cancer Center, School of Nursing, University of Texas Health San Antonio, San Antonio, TX, United States; ^4^School of Public Health, Southeast University, Nanjing, China

**Keywords:** big data, COVID-19, multi-scalar, data-driven, contact tracing, China, COVID-19 exceptionalism

China's COVID-19 exceptionalism is more than just the imposed zero-COVID policies that distinguish its pandemic control approach. It has shifted drastically in a short time, moving beyond just the use of aggregated big data. The positive and negative sides of such data use are already widely debated in scholarly research. As a follow-up, this study discusses how China manages to promptly track and trace every infected case using data-driven governance methods. This approach may seem unfeasible in theory with many difficulties and deficiencies, but it has been effectively practiced. Therefore, it is regarded as China's COVID-19 exceptionalism.

To date, China has shifted from just the basic use of big data to a sort of data-driven governance (1). With big data, the governments and related authorities have access to the individuals' spatio-temporal data. This approach allows tracking all contacts to the infected case in their respective geographic locations and within a certain timeframe. This approach is based on the early introduction of “digital contact tracing” (2, 3), requiring to consider specific ethical guidelines (4) and data justice (5). To improve the effectiveness of digital tract tracing, communities are employed as a moderator in the interactions between government, enterprise, and citizens. In contrast, proactive interactions among key stakeholders in an open innovation ecosystem are identified as the primary contributor (3). Besides community involvement, large-scale public participation also plays a critical role in enhancing public service quality and improving government decision-making effectiveness (3). On the other hand, digital contact tracing promotes the reconciliation of the common interests in e-governance, innovation-driven strategy-making, public-private partnerships, etc. (3).

However, when individual data is used, there are apparent challenges to addressing ethical factors and data justice. It is well-noted that personal information and individual data collected and stored on social networking platforms are vulnerable to data breaches, hacking and scarping, including location information, health information, religious identity, facial recognition imagery, etc. (6). According to the United Nations (7), social media can be one of the main sources of cyber-harassment involving cyberbullying, harassing calls, privacy violations, and so on. In this case, individual-based data is the only effective way forward to increase the accuracy of such detection methods.

Regarding COVID-19 digital contact tracing applications, Shahroz et al. (8) argue that several technological solutions such as drones, blockchain, Artificial Intelligence (AI), etc., have been developed and used by different countries to conduct smart pandemic control and prevention strategies. Generally, timely preventive measures with an integration of both high and low technological solutions worked effectively for some countries like South Korea, China, etc., particularly in supporting contact tracing for surveillance and quarantine (8). India's Aarogya Setu App (translated loosely as “bridge to health”) was dubbed as the arsenal of digital surveillance measures during the pandemic (9). In another study, examining the efficiency of three European contact tracing applications of COVID-19 (StopCovid in France, NHS COVID-19 in the UK, and Coronalert in Belgium), the results show a significant risk of a private data breach in Belgium's Corona-Alert while the UK government switched from NHSX to NHS Covid-19 after only three-month adoption of NHSX due to its low compatibility to share data among smartphones for tracing (10). As for France's StopCovid, Jacob and Lawarèe (10) finds three data security-related risks, including hacking the central database, misreporting fake or unverified positive cases, and increasing the security risks of smartphones as it requires keeping Bluetooth activated.

To summarize briefly, we see changes that are likely to stay as the new normals. The boosted digitalization and digitization in the COVID-19 era (11) means new opportunities for data-based, data analytics, and data-driven methods for better governance and management of the pandemic (12). China's shift from data-driven approaches to multi-scalar contact tracing is an approach that is gone beyond just big data use. At first, larger-scale data were used, and restrictions were imposed at the larger spatial levels of cities and districts. Nevertheless, data use has become smarter and more effective now, allowing for detection and analysis of smaller scales of households, compounds, and neighborhoods. Furthermore, the initial platforms correlated with mobility data that partly relied on self-reporting, leading to new interventions for big data use and data-driven governance (13–15). The evolution of big data use has led to the development of new digital programmes and applications, and such platforms are now further developed based on contact tracing measures, location-based data, and integrated spatio-temporal analysis.

Moreover, it is evident that China's take on a multi-scalar contact tracing approach has evolved drastically. The detected infected person's data is used according to the person's location data, where and when the person has been in contact with others, and how those contacts are then assessed against the larger context of their interactions and exchanges. This approach has enabled a multi-scalar contract tracing method, including at least three levels: (1) person-scale (or individual scale) to evaluate the data of the infected person with their immediate contacts, (2) community-scale to trace and evaluate all contracts with the larger community scale through locational and time data, and (3) zone-scale, to trace a particular boundary or zone where those interactions may have occurred with potential risks under the district level. Therefore, such a multi-scalar approach is a preventive method against the immediate lockdown of districts and cities, which would ultimately reduce the larger-scale disruptions.

Lastly, we highlight the value of big data analytics only at multiple spatial levels, which is more effective for contract tracing. Such an approach cannot happen in isolation (16), and it is only viable to consider in-depth spatio-temporal analysis methods, which are more accurate. A multi-scalar approach would also enhance tracing management (17) to ensure contact tracing is more than just detection and toward containment and reduction of disease spread. The downsides are personal data use, privacy, and ethical factors at the person-scale and inconsistency and incompatibility among different data platforms at the community- and zone-scale, which should be addressed more carefully in future data-driven contact tracing practices. Thus, we urge to consider integrated models for future data-driven contract tracing methods and consider the individuals' privacy and inter-system interoperability.

## Author Contributions

AC drafted the paper. Contributions from TZ, WC, HC, and ZS were received for data collection and reviews. All authors contributed to the article and approved the submitted version.

## Conflict of Interest

The authors declare that the research was conducted in the absence of any commercial or financial relationships that could be construed as a potential conflict of interest.

## Publisher's Note

All claims expressed in this article are solely those of the authors and do not necessarily represent those of their affiliated organizations, or those of the publisher, the editors and the reviewers. Any product that may be evaluated in this article, or claim that may be made by its manufacturer, is not guaranteed or endorsed by the publisher.
